# Zosteriform Cutaneous Metastasis of Lung Adenocarcinoma Mimicking Herpes Zoster: A Diagnostic Pitfall

**DOI:** 10.7759/cureus.114243

**Published:** 2026-08-10

**Authors:** Ela Gazal, Ümit Türsen, Yasemin Yuyucu Karabulut

**Affiliations:** 1 Dermatology, Mersin University Hospital, Mersin, TUR; 2 Pathology, Mersin University Hospital, Mersin, TUR

**Keywords:** cutaneous metastasis, dermatomal eruption, herpes zoster mimic, lung adenocarcinoma, ttf-1, zosteriform

## Abstract

Zosteriform cutaneous metastasis from lung cancer is rare, but dermatomal distribution can lead to diagnostic anchoring on herpes zoster. A 65-year-old man with metastatic lung adenocarcinoma presented with a one-month history of painless, nonvesicular papules confined to the left T7-T9 dermatomes. Herpes zoster was initially suspected because of the striking unilateral dermatomal distribution and the patient's oncologic treatment-related immunosuppression, despite the absence of pain, vesiculation, and crusting. Oral valacyclovir was prescribed. The patient did not attend the planned one-week follow-up and next presented approximately six months later, when the eruption had progressed to extensive infiltrated violaceous papules and plaques spanning T2-T10. Histopathologic examination showed dermal infiltration by malignant gland-forming epithelial cells with lymphovascular invasion, and nuclear thyroid transcription factor-1 positivity supported pulmonary origin. The patient died approximately seven months following the diagnosis of cutaneous metastasis. This case documents the progression of initially limited zosteriform lesions to extensive infiltrative cutaneous metastases. In patients with malignancy, dermatomal distribution should not outweigh discordant morphology or clinical behavior; painless, nonvesicular, persistent, or progressive eruptions warrant early biopsy.

## Introduction

Cutaneous metastases from internal malignancies are uncommon but clinically important because they may indicate advanced systemic disease and can mimic benign, inflammatory, or infectious dermatoses [1]. Lung carcinoma is an important source of cutaneous metastasis in male patients, and skin involvement from lung cancer most often presents as papules or nodules [1,2]. Zosteriform cutaneous metastasis is a rare dermatomal pattern that may be clinically confused with herpes zoster [3-8]. We report a patient with metastatic lung adenocarcinoma who developed a painless, nonvesicular zosteriform eruption initially treated as herpes zoster, emphasizing the importance of early biopsy when the morphology or clinical course is inconsistent with that diagnosis.

## Case presentation

A 65-year-old male patient with metastatic lung adenocarcinoma presented with a unilateral eruption on the left side of the trunk that had been present for approximately one month. Dermatologic examination revealed painless papules arranged in a zosteriform distribution over the left T7-T9 dermatomes on a mildly hyperpigmented background. Vesicles, crusting, ulceration, and subjective symptoms were absent.

The patient had been diagnosed with lung adenocarcinoma approximately 2.5 years earlier, with pleural involvement and metastatic mediastinal, abdominal, and pelvic lymphadenopathy. Because of the advanced-stage disease, he was managed non-surgically. His treatment included radiotherapy and systemic therapy with carboplatin and paclitaxel, followed by nivolumab after disease progression.

Herpes zoster was initially suspected because of the striking unilateral dermatomal distribution and the patient's oncologic treatment-related immunosuppression. However, the absence of pain, vesiculation, and crusting was unusual. A biopsy was not proposed at the initial visit. Oral valacyclovir 1 g three times daily for seven days was prescribed, and follow-up was scheduled after one week to assess the clinical response and reconsider the diagnosis, including biopsy, if the lesions persisted. The patient did not show up for the scheduled follow-up and next presented approximately six months later.

**Figure 1:**
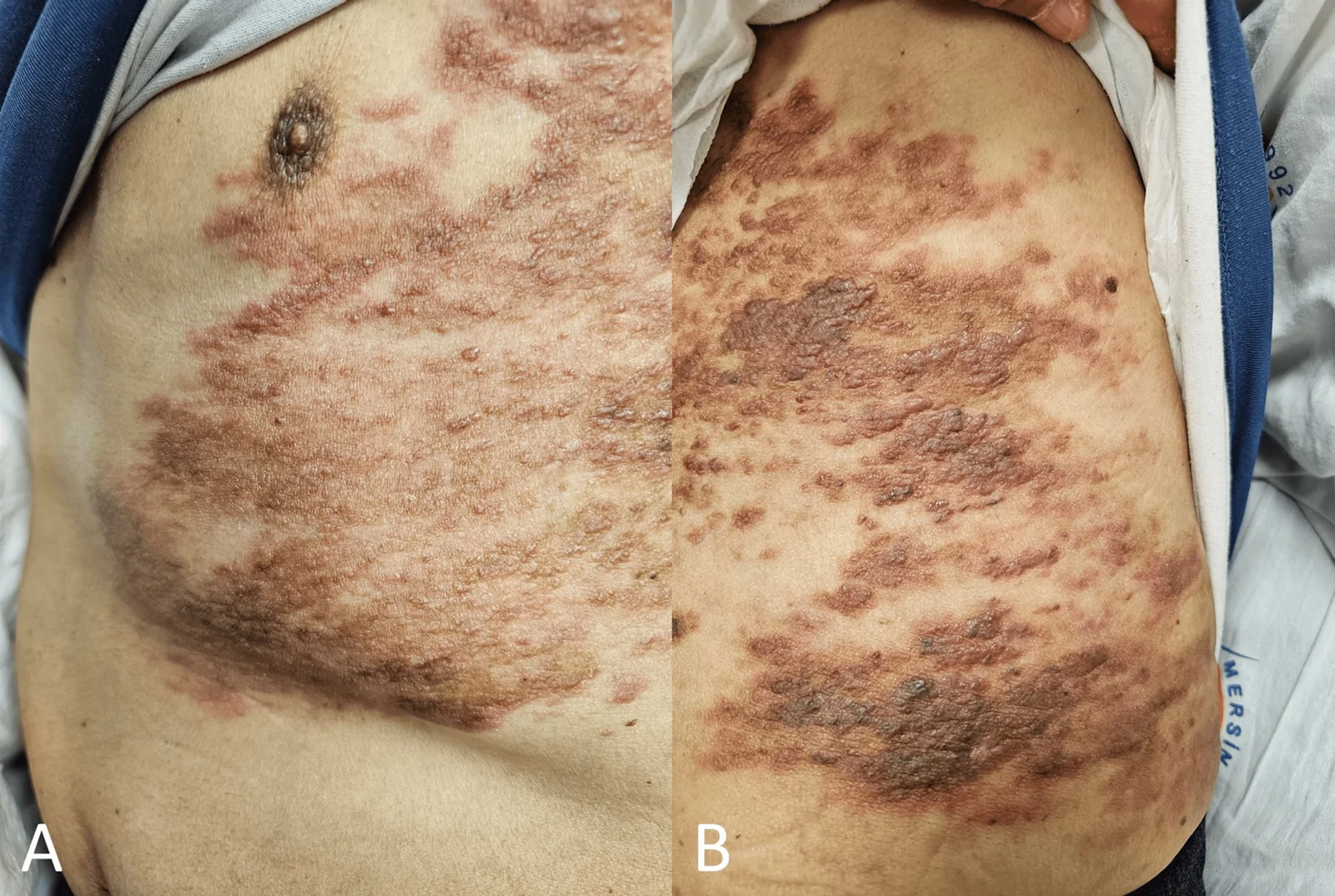
Clinical appearance of the patient's cutaneous metastases at the six-month follow-up. (A) Anterolateral view of the left trunk shows multiple infiltrated violaceous papules in a zosteriform distribution on a prominent hyperpigmented background. (B) Posterolateral view of the same eruption shows greater coalescence of the lesions into infiltrated violaceous plaques, with scattered papules extending toward the back.

At follow-up, the eruption had progressed markedly. The lesions, initially limited to several thoracic dermatomes, had extended over much of the left anterolateral trunk, from approximately T2 to T10. Examination showed infiltrated violaceous papules and plaques with focal coalescence on a hyperpigmented background (Figure 1). The prolonged painless course, absence of vesicles or crusts, progressive extension, and lack of response to antiviral therapy prompted biopsy.

**Figure 2:**
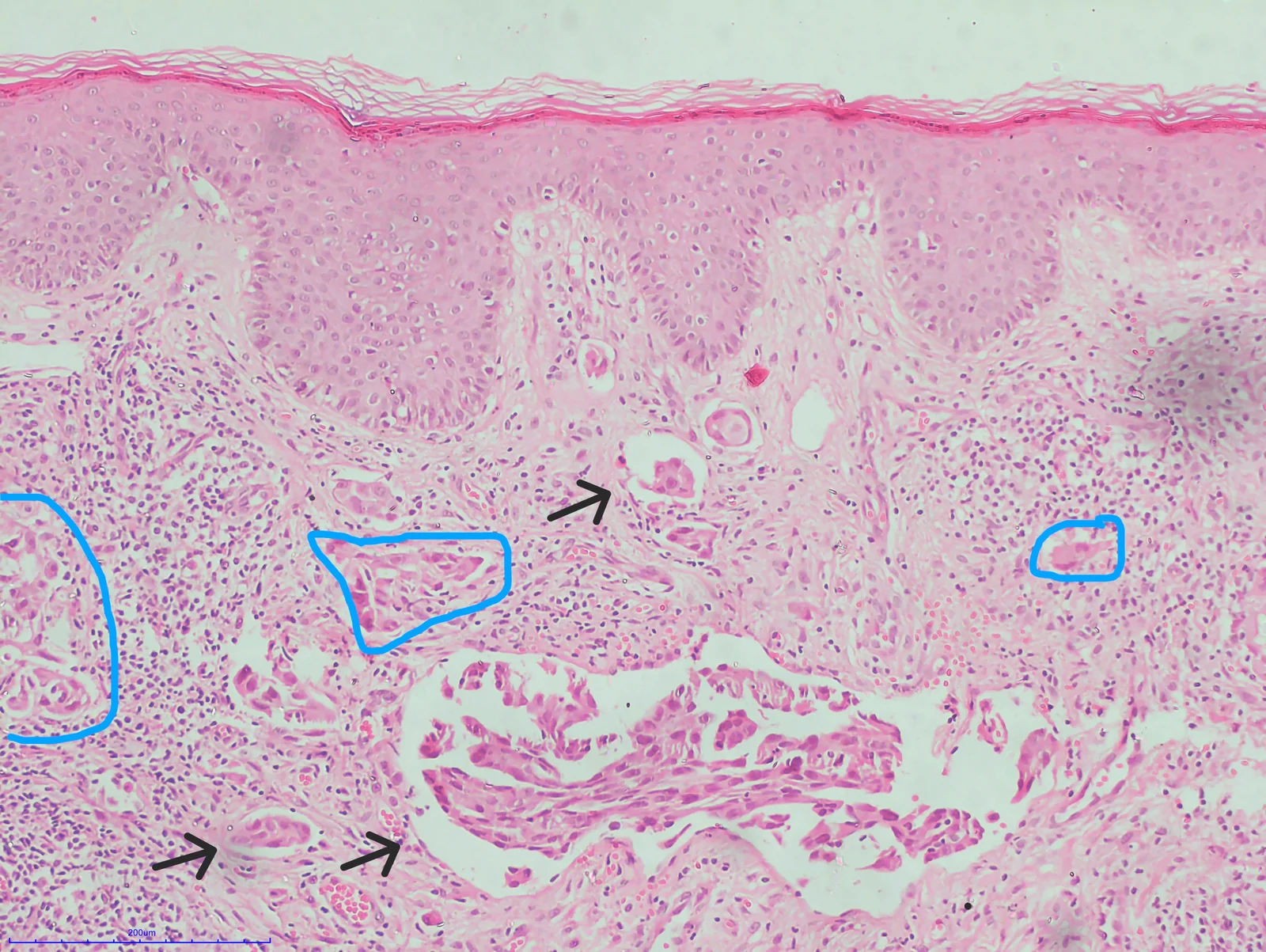
Histopathologic features of zosteriform cutaneous metastasis from lung adenocarcinoma. Punch biopsy specimen from the patient's zosteriform trunk lesions demonstrates dermal infiltration by malignant gland-forming epithelial cells. Blue outlines indicate foci of dermal tumor infiltration, while black arrows indicate tumor emboli within lymphatic spaces, consistent with lymphatic invasion (hematoxylin and eosin, original magnification ×100).

**Figure 3:**
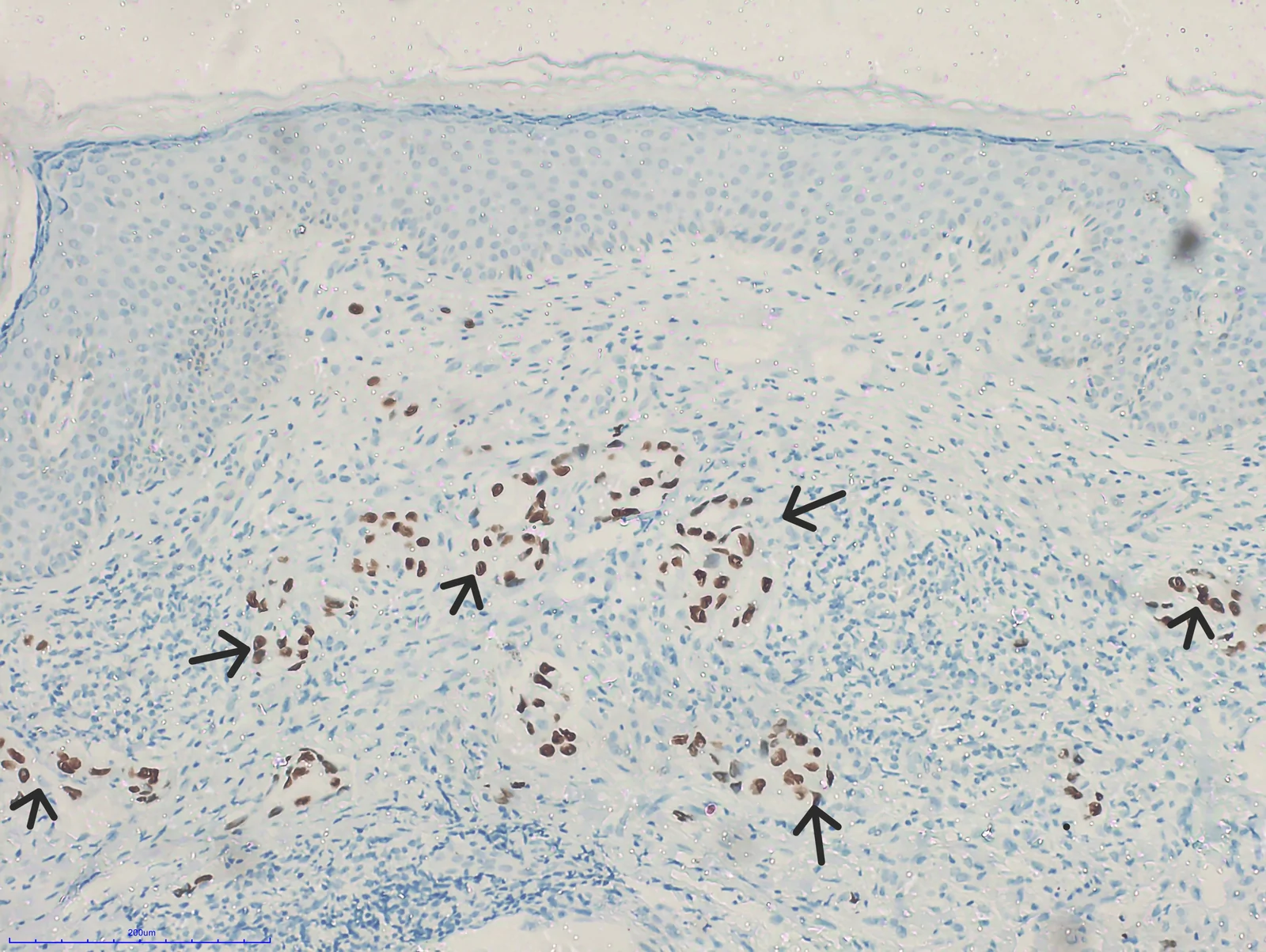
TTF-1 expression supporting pulmonary origin. Immunohistochemical staining of the patient's biopsy specimen demonstrates nuclear TTF-1 positivity in the dermal neoplastic cells. Black arrows indicate representative TTF-1-positive tumor cells, supporting cutaneous metastasis from the patient’s known lung adenocarcinoma (original magnification ×100). TTF-1: thyroid transcription factor-1

Histopathologic examination of a punch biopsy obtained from a representative papule on the left posterolateral trunk revealed dermal infiltration by malignant epithelial cells forming glandular structures, with lymphovascular invasion (Figure 2). Immunohistochemical staining demonstrated nuclear positivity for thyroid transcription factor-1 (TTF-1) (Figure 3), supporting cutaneous metastasis from the known lung adenocarcinoma.

After the diagnosis of cutaneous metastasis, the systemic disease continued to progress and was complicated by recurrent pleural and pericardial effusions and pulmonary emboli. The patient died approximately seven months following the diagnosis of cutaneous metastasis.

## Discussion

Zosteriform cutaneous metastasis is an uncommon presentation of skin involvement by internal malignancy and is defined primarily by its dermatomal or pseudo-dermatomal distribution rather than by a single morphology [3,4]. This pattern has been reported with various primary malignancies, including lung cancer [4]. Published lung cancer cases show considerable variation in lesion morphology, associated symptoms, and initial clinical assessment, as summarized in Table 1 [5-14].

**Table 1:** Selected clinically well-characterized cases of zosteriform cutaneous metastasis from lung cancer. Cases with sufficient patient-level information to compare lesion morphology, subjective symptoms, anatomical distribution, and initial clinical assessment were included. CM: cutaneous metastasis; F: female; HZ: herpes zoster; HSV: herpes simplex virus; M: male; NR: not reported; VZV: varicella-zoster virus

Reference	Patient and primary tumor	Timing relative to lung cancer diagnosis	Cutaneous morphology	Subjective symptoms	Location and distribution	Initial assessment or management
LeSueur et al., 2004 [9]	66/F; bronchogenic adenocarcinoma	Approximately 1 year after diagnosis; shortly after radiotherapy	Multiple red, indurated, crusted papules and nodules with edema and peau d’orange	Initially painless; later painful with leg swelling	Right thigh, extending to the groin, vulva, and suprapubic region in a dermatomal pattern	Diagnosed as HZ; valacyclovir for 3 weeks without improvement
Li et al., 2012 [5]	51/M; right lower-lobe adenocarcinoma	Interval NR	Multiple reddish-to-pink, fixed, firm nodules	Tender	Right upper chest, T3-T5 dermatomes	Initial diagnosis NR; confirmed by skin biopsy
Subramanyam et al., 2016 [10]	75/M; occult right upper-lobe adenocarcinoma	Cutaneous eruption was the presenting manifestation	Erythematous papulonodules with pinhead vesicles, later progressing to fungating and bleeding lesions	Initially painless; subsequently developed mandibular/parotid-region pain, hoarseness, and dysphagia	Anterior chest, later extending to the bilateral neck and right shoulder	Treated as HZ by a local physician; progression prompted biopsy and oncologic evaluation
Aiempanakit et al., 2017 [6]	68/M; lung adenocarcinoma	1 year after diagnosis	Well-defined erythematous indurated plaques containing vesicles, nodules, and crusts	Asymptomatic	Left anterolateral chest, T4 dermatome	No initial HZ treatment reported; biopsied
Wark et al., 2020 [11]	78/M; previously undiagnosed right upper-lobe adenocarcinoma	Cutaneous eruption led to the cancer diagnosis	Erythematous-to-violaceous plaques with microvesicles and dilated capillaries	Nonpainful and nonpruritic	Began around a right internal jugular vascular catheter and spread over the right chest, shoulder, and neck, with a separate right abdominal area; sharp midline demarcation	VZV and HSV swabs were negative; skin and lymph-node biopsies performed
Wang and Xue, 2020 [12]	49/F; lung adenocarcinoma	1 year after diagnosis	Pink-to-red, firm, 0.5-1.0 cm vesicle-like papules	Painful and tender	Left breast in a T4 distribution	Initially diagnosed as HZ at another hospital; antiviral treatment NR
Dagdelen et al., 2020, Case 3 [13]	52/M; right lung carcinoma, subtype NR	51 months after diagnosis	Infiltrated erythematous shiny papules with localized swelling	Asymptomatic	Right chest, T4-T6 dermatomes	Initial diagnosis NR; biopsy performed
Maki et al., 2021 [8]	68/M; right upper-lobe squamous cell carcinoma	Approximately 4 months after diagnosis	Papules	No pain reported	Right anterior and lateral chest, T2-T4 dermatomes	Recognized as disease progression and biopsied; HZ treatment not reported
Tsai and Lo, 2024, Case 1 [7]	72/M; right middle-lobe adenocarcinoma	47 months after diagnosis	Erythematous indurated papules with a vesicular appearance on an erythematous background	Asymptomatic	Right trunk, T6-T8 dermatomes	Initial HZ diagnosis or treatment not reported; biopsied
Tsai and Lo, 2024, Case 2 [7]	91/M; previously undiagnosed lung adenocarcinoma	Diagnosed simultaneously with CM	Pink-reddish, vegetative, shiny, pinhead-sized papules forming confluent plaques	Asymptomatic skin lump	Right anterior chest, T1-T4 dermatomes	Initial HZ diagnosis or treatment not reported; biopsy established the diagnosis
El Bakkali et al., 2026 [14]	62/M; previously undiagnosed right pulmonary adenocarcinoma	Diagnosed simultaneously with CM	Multiple firm, fixed, erythematous-to-violaceous nodules, some ulcerated with erosion and crusting	Cutaneous symptoms NR	Linear distribution over the right T3-T6 dermatomes	Initially treated as HZ with antiviral therapy; no improvement
Present case	65/M; metastatic lung adenocarcinoma	Approximately 2.5 years after lung cancer diagnosis	Initially nonvesicular papules on a hyperpigmented background; later infiltrated violaceous papules and plaques	Painless; no associated subjective symptoms	Initially left T7-T9, subsequently extending from T2 to T10	HZ suspected; valacyclovir 1 g three times daily for 7 days; scheduled one-week follow-up not attended

The present case adds to this literature by focusing on the diagnostic decision point rather than the distribution alone. Herpes zoster typically evolves from macules and papules into vesicles and pustules over several days and is often accompanied by pain or sensory symptoms [15]. In this patient, the eruption was dermatomal but painless and nonvesicular at presentation. The clinical pitfall was that the striking zosteriform arrangement outweighed these discordant features and led to an initial diagnosis of herpes zoster.

Although empiric antiviral therapy was reasonable initially, given the patient's oncologic background and dermatomal eruption, diagnostic anchoring at the initial visit and the missed scheduled follow-up contributed to the delayed diagnosis. By the time of re-evaluation, the lesions had become more extensive and infiltrated, and the lack of response to antiviral therapy made a viral process less likely. This sequence supports a low threshold for biopsy in patients with malignancy when a presumed zosteriform eruption is nonvesicular, painless, persistent, progressive, or treatment-refractory.

Histopathology confirmed the diagnosis by demonstrating dermal infiltration by gland-forming malignant epithelial cells, while TTF-1 positivity supported pulmonary origin [16]. The patient's systemic disease subsequently progressed, with recurrent pleural and pericardial effusions and pulmonary emboli. The patient was reported deceased approximately seven months following the diagnosis of cutaneous metastasis, consistent with the poor prognosis generally associated with cutaneous metastasis from lung cancer [1,2].

The mechanism of zosteriform metastasis remains uncertain. Proposed explanations include lymphatic spread, perineural dissemination, hematogenous spread, and localization to previously altered skin [3,4,8]. A previous lung cancer case demonstrated retrograde lymphatic migration through radiologic and autopsy findings [8]. In the present case, lymphovascular invasion raises the possibility of lymphatic spread; however, its relationship to the zosteriform distribution cannot be established from a single skin biopsy. Regardless of the underlying mechanism, a dermatomal eruption in a patient with malignancy should not be assumed to represent herpes zoster solely on the basis of its distribution. Discordant morphology, persistence, progression, or failure to respond to antiviral therapy should prompt early biopsy.

## Conclusions

Zosteriform cutaneous metastasis should be considered when a patient with malignancy develops a dermatomal eruption, even when treatment-related immunosuppression makes herpes zoster appear more likely. The absence of pain and vesiculation, together with persistence, progressive infiltration, and failure to respond to antiviral therapy, should prompt early biopsy.
